# Subbandage pressure assessment in the lower limb during supine positioning of patients in hip arthroplasty when traction is applied: a comparison between single and double layer bandages

**DOI:** 10.1007/s00402-025-06106-1

**Published:** 2025-10-24

**Authors:** David Putzer, Ulyana Konopada, Johannes Domenikus Pallua, Rohit Arora, Michael Nogler

**Affiliations:** 1 Department of Orthopaedics and Traumatology, Experimental Orthopaedics, Innsbruck Medical University, Innsbruck, Austria; 2 Department of Mechanical and Aerospace Engineering, Polytechnic University of Turin, Turin, Italy; 3 Department of Orthopaedics and Traumatology, Innsbruck Medical University, Innsbruck, Austria

**Keywords:** Patient positioning, Subbandage pressure, Traction table, Joint dislocation, Bandage systems

## Abstract

**Purpose:**

Patient positioning in orthopedic and trauma surgery requires maximum stability while preserving passive mobility of the operated limb. When traction tables or positioning aids are used, the legs are secured in traction boots. This study evaluated sub-bandage pressure in the lower extremity of a supine-positioned specimen during hip arthroplasty, comparing single- and double-layer bandage systems.

**Methods:**

A flexible multilayer and a more rigid single-layer bandage system were compared in mechanical traction tests on a cadaveric specimen. Subbandage pressure was recorded through repetitive measurements at various traction forces, ranging from 80 to 200 N.

**Results:**

Due to its rigidity, iFix generated higher pressure near the ankle, providing better stability even at 200 N. In contrast, CO showed higher pressure at the proximal tibia and heel lift at maximum force due to its elasticity. The study found that patient fixation using the tested systems is only justifiable if the tensile force remains below 80 N throughout surgery.

**Conclusion:**

Fixation with the tested systems is only advisable if the tensile force remains below 80 N throughout surgery. Short-term increases (e.g., for hip dislocation) are acceptable but should be brief and followed by adequate relief to allow tissue reperfusion. Additional padding is strongly recommended to distribute subbandage pressure at high traction forces.

## Introduction

Total Knee Arthroplasty (TKA) and Total Hip Arthroplasty (THA) rank among the most widely performed surgical procedures worldwide: almost 2.8 million cases are performed annually, with the number increasing every year [12]. Even more striking is the expected growth of revision surgery in the lower limbs, which in just a decade will demand will be almost comparable to that of primary implants, reaching rates of + 173% and even + 601% (for THA and TKA revision, respectively) [13].

Proper patient positioning during surgery is essential to ensure optimal access to the affected area. Incorrect positioning can lead to serious complications, such as joint dislocations, nerve damage, muscle pain, and deep tissue injuries to the skin [3]. Positioning systems must be capable of withstanding significant mechanical forces - for example, those exerted during joint dislocation or hip implant placement - while also stabilizing the patient to prevent unintended movement [8, 24]. Various positioning techniques have been described in the literature, each tailored to the specific surgical site and objective [2].

Several patient positioning methods exist based on the surgical approach to the hip joint and surgeon preference. The dorsal approach must be performed in a lateral position. The direct lateral and anterolateral approaches can be performed in either a lateral or supine position, whereas the direct anterior approach must be performed in a supine position [22]. Positioning of the patient’s leg can be achieved with or without the help of a traction table or leg support [16, 19, 27]. Manoeuvrability of the leg is essential to achieve good exposure to the femoral canal [17]. Given that traction tables are not universally available and cost from $100,000 to $200,000 + CAD (Canadian Dollars) [27], several specific table attachments are available as well [9, 16, 28]. Additionally, the standard table technique may offer perioperative advantages, including decreased blood loss, shorter operative time, and fewer intraoperative fractures, compared to traction tables [27].

The correct and secure positioning of the patient during surgery is crucial. Not only should patient positioning allow for appropriate access to the operative area by the surgeon, but it should also prevent lesions such as nerve damage or impaired circulation [3] and prevent unintended positional changes in the patient due to the simultaneous presence of passive mobility in the operated extremity. Additionally, patient positioning should not represent a physical burden beyond measure for the staff.

The most common reason for revision in hip arthroplasty is the loosening of the prosthesis [30]. Patient-related factors may cause this, including the surgeon’s experience with the operation or the implant itself [5]. Apart from that, the outcome highly depends on the accurate positioning of the acetabular cup [7]. Padgett et al. stated that even if the same surgeon performs multiple hip prosthesis implantations, the angle of the acetabular cup can vary quite a bit (23°-57 ° angle of abduction) [20]. According to Padgett et al. and Brodt et al., this can be associated with the patient’s hip movements during surgery, induced by high forces [4, 20].

Complications related to patient positioning include skin pressure sores, nerve compressions, deep vein thrombosis (DVT) and compartment syndrome [6]. Skin pressure sores can lead to sepsis, which can be fatal. Stevens et al. have demonstrated that patients with longer operative times are at a greater risk of skin breakdown and require more intensive care during preoperative positioning [29]. Theatre staff should ensure that pressure points around bony prominences, such as the iliac crest, sacrum, and heels, are well-padded with pillows, ankle pads, etc. If necessary, the patient should be moved slightly during lengthy procedures. Ideally, the pads and pillows should ‘absorb compressive force, redistribute pressure, prevent excessive stretching and provide support for optimum stability’ [26]. Theatre staff must be aware of the potential risk of nerve injury in patients with poor positioning [6]. Rothrock noted that neurological complications can arise within minutes, but they have long-lasting effects on the patient [26]. Common peroneal nerve damage results in a foot drop and a loss of sensation over the lateral leg and dorsum of the foot [31]. DVT can result from prolonged periods of immobility, causing blood stasis, and is associated with the potential life-threatening complication of the pulmonary embolic phenomenon [14]. Compartment syndrome is a life-and limb-threatening complication associated with patient mal-positioning [15]. Safe positioning must allow for adequate blood flow to all four limbs.

To secure the lower extremity to the traction boots, various types of bandages can be used. The tighter the fixation to the traction boot, the more efficient and accurate the traction will be. On the one hand, higher traction could allow for intra-articular manoeuvrability and reduce the risk of intra-articular complications and damage to articular cartilage; on the other hand, it can cause nerve and soft tissue injuries [10]. Bandages should apply enough pressure to secure the lower extremity in the traction boot while minimizing pressure to prevent pressure-related injuries and ensure effective traction force transmission.

In this study, the effect of traction forces during supine patient positioning on subbandage pressure was evaluated in three different zones of the lower leg when using single- and double-layer bandage systems for patient stabilisation.

## Materials and methods

Two different bandage systems were evaluated for patient positioning on the lower limb when being used on a traction table for hip arthroplasty. To fixate the patient, a carbon foot attachment was used (Stryker, Kalamazoo, MI, United States). Coban^TM^ 3M^TM^ (CO) (3M Health Care, St. Paul, MN, United States) is a self-adherent elastic wrap made from natural rubber latex, available in both sterile and non-sterile versions. CO is used to secure dressings and other devices, compress or protect wound sites, and immobilize injuries.

iFIX (iSYS Medizintechnik GmbH, Kitzbühel, Austria) is a non-sterile product intended for patient stabilisation over a short period, during both invasive and non-invasive procedures, by securing the position of the patient’s anatomy with intact skin outside the sterile field. The iFIX system consists of two parts: the iFIX Fleece (the bandage) and the iFIX Patch. The stretchy fleece is a single-use component. Depending on the table design, an iFIX Patch that adheres to the table surface, an iFIX Slot Adapter that slides into the channels, or an iFIX Patch Adapter that connects to standard hook fasteners can be chosen to secure the patient to the table. The micro anchors of the iFIX Patches and Adapters provide strong security for the iFIX Fleece, with a peel strength of 2300 N/m and a shear strength of 10,000 N/m, as specified by the manufacturer. iFIX Fleece is made of nonwoven polypropylene fleece and has a thickness of 0.83 mm. The iFIX Patch is composed of polyamide extruded with artificial rubber, providing humidity and wet resistance.

**Fig. 1:**
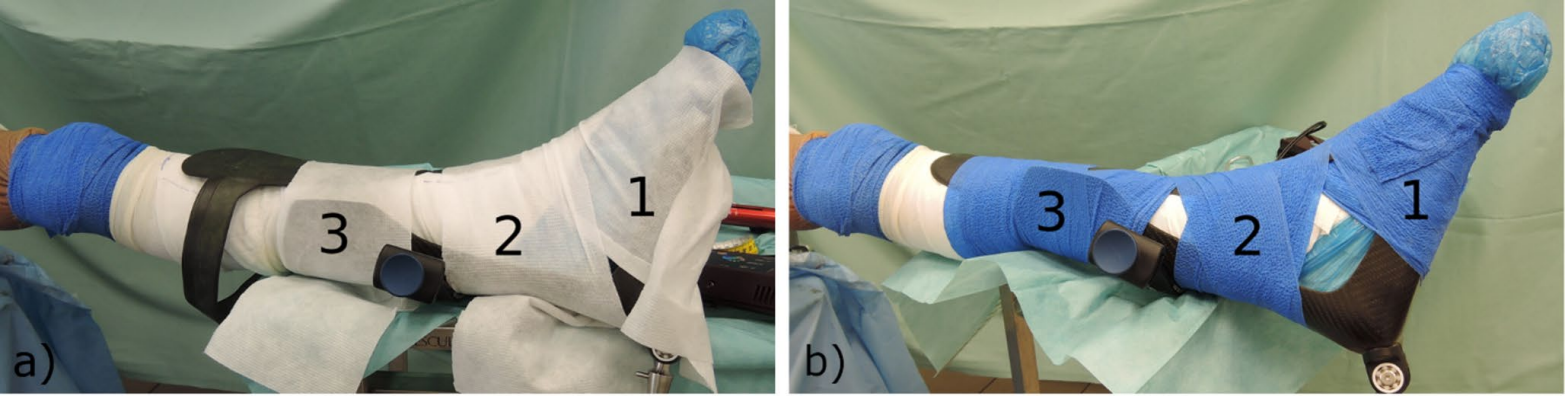
Configuration of the bandage system on the lower extremity using a single layer of iFIX fleece (**a**) and a double layer of CO (blue) (**b**) in three zones: (1) foot, (2) proximity of the ankle (3) shank

Subbandage pressure measurements were carried out on a cadaveric specimen with a leg length of 78 cm (measured from the heel to the estimated hip rotation centre). Informed consent was obtained for the donation of the body for research purposes. The specimen was positioned supine on a table with the leg in full extension and secured with a body strap around the upper body. The foot of each specimen was placed in a rigid footrest (boot) with a foam pad and secured at three points with either a double layer of CO or a single layer of iFIX fleece. The iFIX fleece was attached to iFIX patches, which were secured to the boot itself in three different zones (foot, ankle, and shank), as shown in Fig. 1a. To safely fit the specimen’s leg in the boot iFIX fleece was cut in three pieces with 20 × 50 cm for the foot (1), 16 × 50 cm for the distal tibia part (2) of the leg, 12 × 50 cm for the proximal tibia (3) (Fig. 1a) ).

CO (10 cm x 4,5 m) has adhesive properties between different layers (it doesn’t need the patch), and the foot was fixed with two rounds of CO in each zone to the boot (Fig. 1b) ).

A dynamometer (measurement range: 0–22 kg, accuracy: ±1 kg) was attached to the heel of the boot, and increasing traction forces were applied at the heel of the foot by pulling the leg distally.

To measure subbandage pressure Two measurement pads (pedar^®^ insoles, novel GmbH, Munich, Germany, thickness 1.9 mm, 99 sensors, pressure range 15-600 kPa, hysteresis: < 7%, Resolution: 2.5 kPa, minimal bending radius 20 mm) were placed between bandage and specimens forefoot as well as between bandage and anterior part of the shank (Fig. 1).

The pressure device should be positioned between the skin and the bandage; however, the specific location of the sensor is a matter of controversy [21]. The primary guideline is not to measure pressure over bony prominences or tendons, as the hardness of the structure can influence the measurement. The curvature of the leg at the sensor position and how the circumference changes during movement must be considered [21]. The minimum recorded pressure was 15 MPa.

An increasing load (80–200 N with 40 N incremental steps) was applied to measure subbandage pressure. Ten measurement repetitions were performed for each bandage, changing the fixation with the two different bandage systems each time. The sample frequency was set to 50 Hz, and the duration of each measurement was 6 s for each load case.

Mean and maximum pressure maps were generated using MATLAB R2024b (The MathWorks® Inc., Natick, MA, USA).

Three different zones were defined to determine the subbandage pressure with the ankle as a reference point at 13,5 cm:

- Foot (F): 0–13,5 cm was the length of the sensors covering the forefoot, width 8,4 cm.
- Distal Tibia (DT): 13.5–25 cm, the sensors were in proximity of the angle joint covering the lower tibia.
- Proximal Tibia (PT): 25–46 cm is the length of the sensors covering the upper tibia.

### Statistical analysis

Mean, median, range, and standard deviation were calculated for the different measurement parameters. For the analysis, GraphPad® Prism (Version 10.2.3, GraphPad Software, Inc., La Jolla, US-CA) was used.

All data were tested for normal distribution using the Kolmogorov-Smirnov test. The two groups were compared with each other using the T-test in cases of normal distribution and the Mann-Whitney U-test in cases of non-normal distribution. A p-value of 0.05 was considered statistically significant.

All boxplots report mean and standard deviation (box) and minimum and maximum (whiskers).

## Results

Although most of the groups reached a normal distribution, there were still some exceptions; therefore, the Mann-Whitney U-test was used in all cases.

**Fig. 2:**
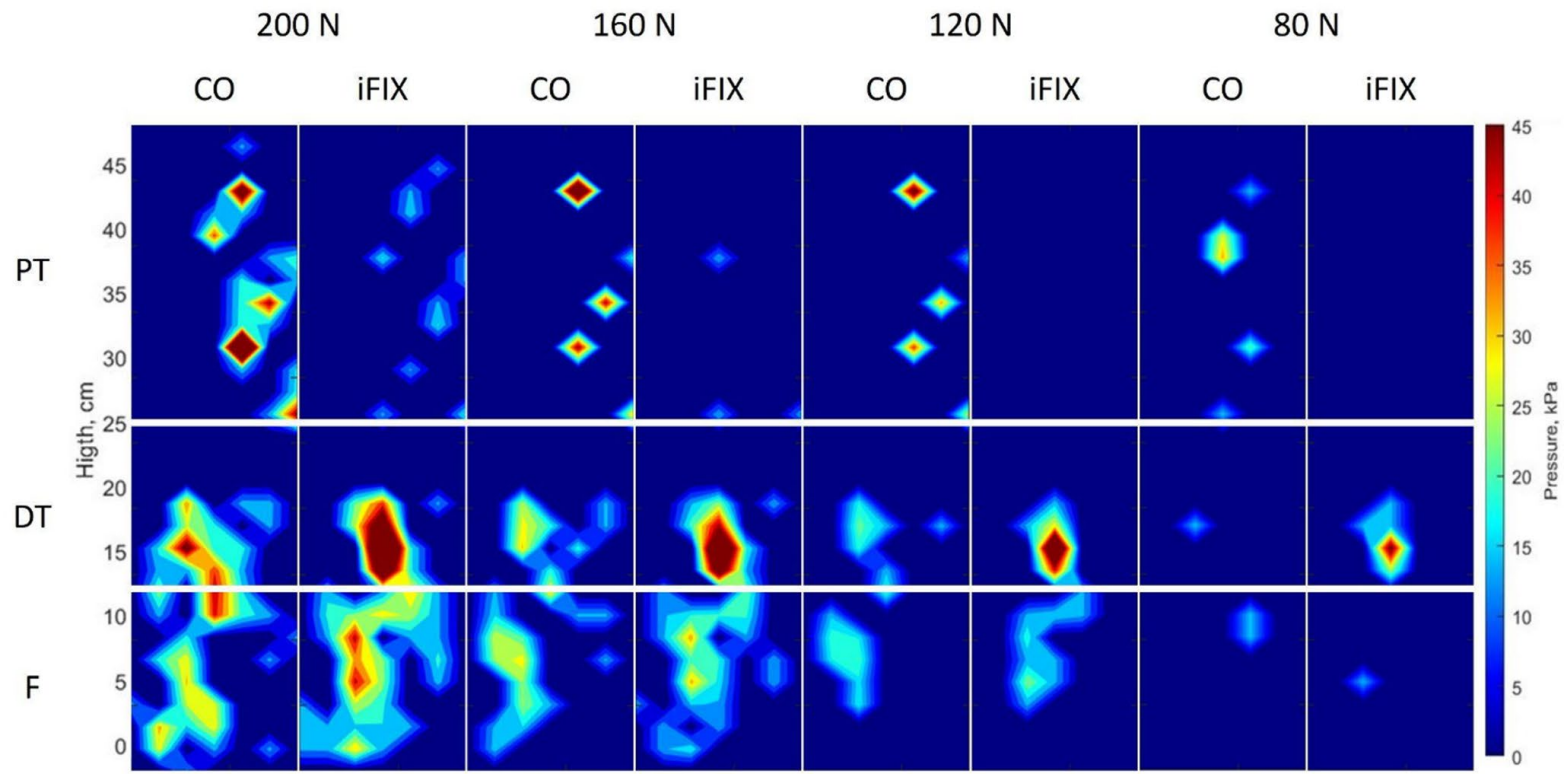
Subbandage pressure maps of the lower extremities reporting the maximum pressure averaged over time for the different loads applied, where the global maximum occured. Red areas indicate zones with high stress, while blue areas show areas with low pressure

**Fig. 3:**
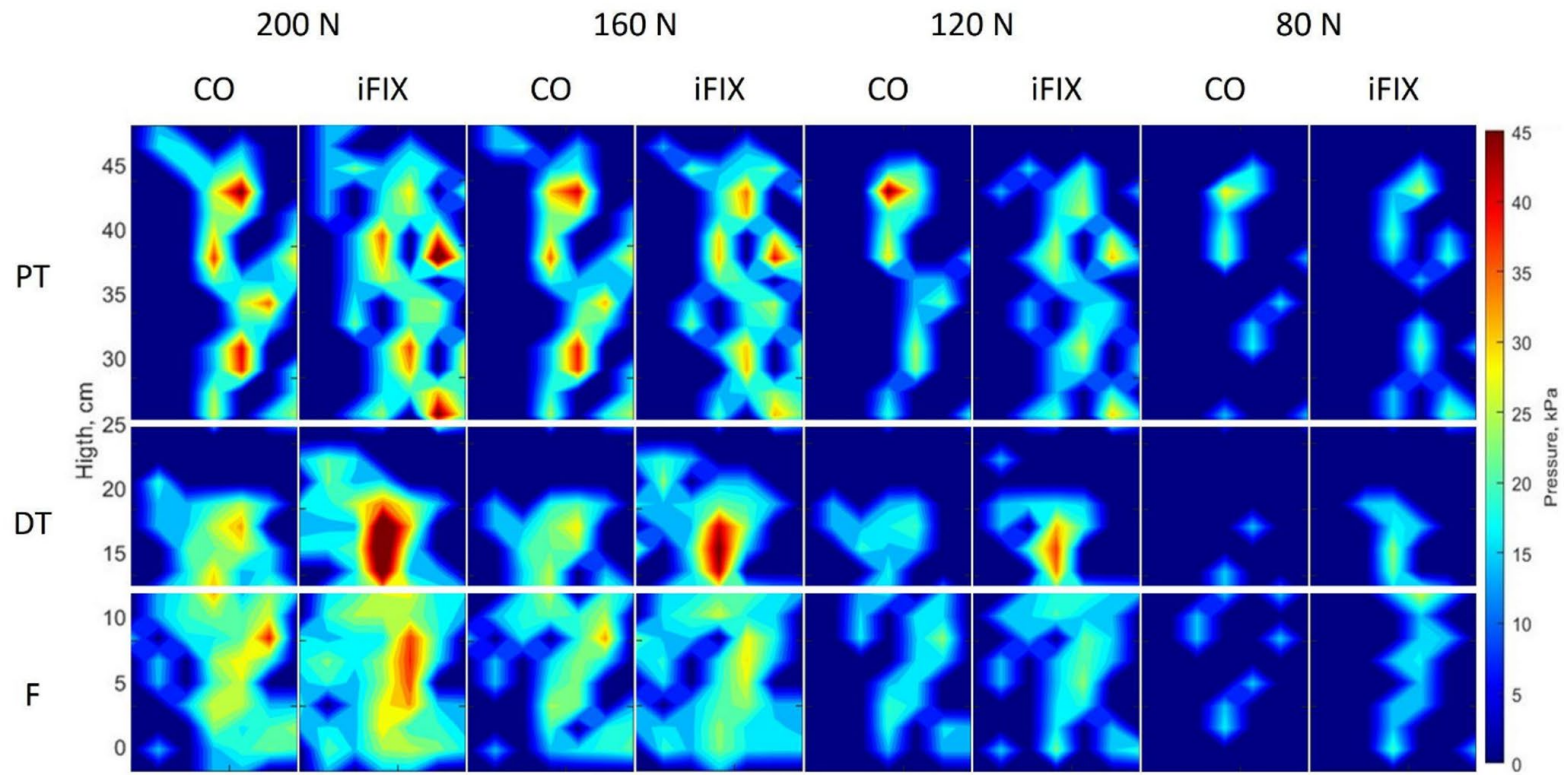
Subbandage pressure maps of the lower extremities reporting the mean pressure averaged over the 10 trials over time for the different loads applied. Red areas indicate zones with high stress, while blue areas show zones with low pressure

When considering the maximum pressure averaged over time, no statistically significant difference could be found for the lower extremity between the iFIX and CO bandage systems (*p* > 0.05, Table 1; Figs. 2 and 3).

On the food region no statistically significant difference could be found between iFIX and CO bandage for all four load levels as well as in the proximal region (*p* > 0.05) except for the load level of 200 N where CO showed a statistically significant higher value (29% higher) than iFIX (*p* = 0.045) (see Table 1; Figs. 2 and 3).

When considering the distal tibia region, a statistically significant higher maximum pressure was found for all four load levels for the iFIX bandage compared to the CO group. iFIX showed a 33% higher maximum pressure than CO when loaded with 200 N (*p* = 0.001). For the load level of 160 N, the difference between the two bandage systems was 33% (*p* = 0.001), and for the load level of 120 N, the difference assessed was 39% (*p* = 0.001). At a load level of 80 N, iFIX showed a 36% higher maximum pressure than CO (*p* < 0.001) within the distal tibia region (see Table 1; Figs. 2 and 3).

**Table 1:** Median of the maximum subbandage pressure averaged in time for the lower extremity and divided by the three zones of interest

Zone	Applied force [*N*]	iFIX Median of Maximum Pressure (Range) [MPa]	CO Median of Maximum Pressure (Range) [MPa]	*P*-value
lower extremity	80	30 (20–49)	23 (17–43)	0.183
	120	48 (25–63)	45 (28–60)	0.630
	160	57 (30–79)	55 (42–80)	0.853
	200	61 (40–86)	65 (49–91)	0.481
PT	80	28 (15–38)	21 (15–43)	0.634
	120	32 (21–56)	41 (25–60)	0.356
	160	38 (15–67)	53 (32–80)	0.063
	200	46 (18–76)	65 (35–91)	0.045
DT	80	28 (20–49)	18 (15–23)	< 0.001
	120	41 (20–63)	25 (20–34)	0.001
	160	49 (25–79)	33 (25–43)	0.001
	200	57 (25–86)	40 (32–50)	0.001
F	80	20 (15–32)	19 (15–26)	0.108
	120	30 (20–35)	27 (20–42)	0.280
	160	38 (25–43)	35 (27–53)	0.782
	200	42 (31–49)	43 (33–63)	0.579

Maximum averaged pressure distribution plots reveal a difference in patterns and demonstrate the variation in the DT region between the two groups. The DT seems to be the zone where subbandage pressure appears to be highest for IFix, while CO shows a higher subbandage pressure on the PT zone in proximity to the bony structures (tibia, tibial tuberosity) (see Fig. 2).

The same behaviour, although less evident, can also be observed when considering the average pressure averaged over time. In the DT region, the subbandage pressure shows a larger red zone for IFix in compared to CO. The differences in the upper tibia and lower tibia are becoming less evident when considering the averaged values (see Fig. 3).

A statistically significant difference was found for CO in the distribution between proximal, distal tibia and foot was found for the load case of 200 N (*p* = 0.0115), for the load case 160 N (*p* = 0.006), for the load case of 120 N (*p* = 0.026) while no statistically difference could be found for the load case of 80 N (*p* = 0.615) when considering the averaged maximum pressure. In CO, a statistically significantly higher maximum pressure value could be found for the proximal tibia in comparison to the distal tibia for the load level 200 N (38%, *p* = 0.001), 160 N (38%, *p* = 0.001) and 120 N (39%, *p* = 0.042). Additionally, for a load level of 160 N of CO, a statistically significant higher subbandage pressure was found for the proximal tibia (34% more) compared to the foot region (*p* = 0.042).

iFIX showed a statistically significant difference in the maximum averaged pressure distribution between foot, proximal and distal tibia for the load level 200 N (*p* = 0.030) and 160 N (*p* = 0.046), where a statistically significant higher pressure zone was found for the distal tibia in comparison to the foot region (26% *p* = 0.022) at 200 N and at 160 N (22%, *p* = 0.042). No statistically significant difference in the pressure distribution of iFIX was found for the other two load levels (120 N: *p* = 0.107, 80 N: *p* = 0.524).

## Discussion

The maximum measured subbandage pressure was 65 kPa for CO, with no difference from the iFIX subbandage pressure, which was 61 kPa for the lower extremity when a pulling force of 200 N was applied. The skin vessels of a healthy adult typically exhibit an arterial systolic blood pressure of approximately 80 mmHg (10.64 kPa). This means that any pressure applied to the skin exceeding this value can interrupt blood flow, and if sustained for an extended period, may lead to tissue damage [1]. When observing the distribution of the subbandage pressure in the color maps of the maximum and mean subbandage pressure (Figs. 2 and 3), it is evident that the pressure exerted by both fixation systems are exceeding the threshold of 10.64 kPa already at the lowest load level of 80 N in almost all areas of contact.

In Table 2, acceptable values for subbandage pressure for medical compression hosiery are shown according to the quality assurance standard RAL-GZ 387/1 [18].

**Table 2:** Pressure value at the ankle taken from the quality assurance RAL-GZ 387/1 [18]

Compression class	Compression intensity	Compression in kPa
I	Low	2.4 to 2.8
II	Moderate	3.1 to 4.3
III	High	4.5 to 6.1
IV	very high	6.5 and higher

The compression values of the RAL-GZ 387/1 standard set the security limits for long-term use, to prevent harm to the vascular system [18].

The colour maps in Fig. 1 show a clear difference in the distribution of subbandage pressure for both groups. iFIX shows statistically significantly lower maximum subbandage pressures in the proximal tibia than CO, while CO shows a statistically significantly lower subbandage pressure in the proximal part of the tibia. The highest values for subbandage pressure are found for CO at the proximal tibia, while for iFIX, they are found on the distal tibia. In the foot region, both bandage systems show no difference between each other.

The differing behaviour of the two fixation systems in terms of the location of the most significant pressure load on the leg could be explained by the different material properties of the bandages, as well as how they were secured in the experimental setup. While iFIX consists of a relatively inelastic fleece, the Coban bandage has increased stretchability [23]. Additionally, in the experimental setup, three separate fleece strips of the iFIX system were used, each attached individually (single layer) (see Sect. 2.3). In contrast, the Coban bandage was wrapped around the leg in a multilayer configuration.

The higher elasticity of the CO was observed during the measurements when the pulling force was applied. Indeed, at a maximum traction force of 200 N, a heel lifting up to 2–3 cm was observed. iFIX, therefore, provides a more stable fixation of the leg, which results in higher force in the distal part of the tibia. While on one side this leads to a higher load transfer on the bony areas of the ankle, the more rigid fixation of the leg might, on the other hand, help reduce the traction force, as the load transfer is more efficient. On the other hand, with CO, higher pulling forces might be compensated by the elastic behaviour of the bandages, although this provides some stress points on the proximal tibia. Additional foam pads might be added to protect the bony areas.

Traction forces during surgery may be significantly higher than our highest measured traction force of 200 N. Indeed, Yin et al. reported a mean traction force of 531.8 N exercised on the lower extremity during hip arthroscopy surgery [32]. Röling et al. reported a mean traction force of 714 N (range, 390–1362 N) and a median joint space widening of 8.8 mm for peroperative hip dislocation in hip arthroscopy [25]. The values of traction force measured in the Gao et al. study [11] are slightly lower than those reported in the aforementioned studies: 506.7 N (range, 331.5–759). The traction force should be applied as short as possible, as the generated subbandage pressure of a holding device might cause damage to the tissue and circulatory system in the human leg. The measured subbandage pressures might be even higher in a surgical case.

By exercising a pulling force on the lower extremity, the soft tissue must counterbalance the applied forces. Special care should be taken when traction forces are used, as this might lead to soft tissue damage [10].

### Limitations

The following limitations have to be considered for this study. All measurements were performed on a cadaveric specimen. Due to the conservation method, there may be significant differences in the mechanical properties of the soft tissue, which may not accurately reflect the surgical case of relaxed soft tissue. However, when measuring subbandage pressure, the influence of the soft tissue properties itself might have a less substantial impact on the determined pressure values. When muscle tension is applied, the subbandage pressure may differ significantly from the measured values, as the distribution pattern may change. The surface measured was not covering the whole region of interest; however, they were applied in the areas where we expected the highest subbandage pressure. The pressure generated by the boot itself on the soft tissue is therefore unknown. Bandages were applied by medical personnel, who paid attention to placing them in a similar location. The positioning of the bandages may vary between measurements; however, the exact location was not controlled intentionally to simulate a real case scenario.

Both bandage systems were applied in a dry environment; the material properties might change when soaked in liquids during surgery. However, a previous study showed that iFIX did not alter significantly its properties when in contact with water [23]. In our setup, a constant static pulling force was applied to the specimen. Subbandage pressure may be significantly higher when the specimen is subjected to a dynamic load cycle.

## Conclusion

Thus, this study found that securing a patient to the OR table using the tested fixation systems would only be justifiable if the maximum tensile force remained below 80 N for the entire duration of the operation. Temporarily, the tensile force may be increased for short actions (e.g., during the dislocation of the hip joint), but this should be performed as quickly as possible, followed by a complete and sufficiently long period of relief to allow adequate tissue reperfusion.

The mechanical properties of iFIX caused higher pressure near the ankle because the bandage was more rigid. This ensures better stability even when a maximum force of 200 N is applied. CO showed a higher subbandage pressure on the proximal part of the tibia, and with maximum force applied, also exhibited heel lifting due to its elastic properties.

The location where the fixation system is applied also plays an important role. Thin tissue layers with low subcutaneous fat or superficial bony structures (e.g., bones) are susceptible to pressure loads (see pressure ulcers in the sacral region [31]). The influence of additional padding for the fixation systems in reducing pressure loads as well as its practical use and handling in the operation room should therefore be investigated in a further study. However, we highly recommend adding additional padding to the lower extremity when prolonged exposure to pulling forces above 80 N is expected.

## Data Availability

Data is stored on internal servers and handled out on request.
